# Effectiveness of Hyaluronic Acid Injection in the Reconstruction of Interdental Papilla: A Systematic Review

**DOI:** 10.3290/j.ohpd.c_2057

**Published:** 2025-06-03

**Authors:** Alexia Larderet, Catherine Petit, Olivier Huck, Pierre-Yves Gegout

**Affiliations:** a Alexia Larderet Periodontist, Université de Strasbourg, Faculté de Chirurgie Dentaire, Parodontologie, Strasbourg, France; Hôpitaux Universitaires de Strasbourg, Pôle de médecine et chirurgie bucco-dentaires, Strasbourg, France. Formal analysis, data curation, wrote original draft, read and agreed to the published version of the manuscript.; b Catherine Petit Associate Professor, Université de Strasbourg, Faculté de Chirurgie Dentaire, Parodontologie, Strasbourg, France; Hôpitaux Universitaires de Strasbourg, Pôle de médecine et chirurgie bucco-dentaires, Strasbourg, France. Reviewed and edited the manuscript, read and agreed to the published version of the manuscript.; c Olivier Huck Professor, Université de Strasbourg, Faculté de Chirurgie Dentaire, Parodontologie, Strasbourg, France; Hôpitaux Universitaires de Strasbourg, Pôle de médecine et chirurgie bucco-dentaires, Strasbourg, France. Conceptualisation, methodology, validation, reviewed and edited the manuscript, supervised the study, read and agreed to the published version of the manuscript.; d Pierre-Yves Gegout Associate Professor, Université de Strasbourg, Faculté de Chirurgie Dentaire, Parodontologie, Strasbourg, France; Hôpitaux Universitaires de Strasbourg, Pôle de médecine et chirurgie bucco-dentaires, Strasbourg, France. Formal analysis, data curation, wrote original draft, supervised the study, read and agreed to the published version of the manuscript.

**Keywords:** black triangle, esthetic, gingiva, hyaluronic acide, periodontitis, regeneration.

## Abstract

**Purpose:**

The loss of interdental papilla (IDP) is a significant esthetic concern often associated with black triangles (BT). BT are potential consequences of periodontitis, orthodontic treatment, and anatomical variations due to their influence on the critical distance from the contact point to the bone crest. Various treatment options, both invasive and non-invasive, have been proposed to address this issue. Recently, the injection of hyaluronic acid (HA) has emerged as a promising minimally invasive alternative. This systematic review aims to evaluate the effectiveness of HA injections for IDP reconstruction in esthetic zones in humans.

**Materials and Methods:**

A comprehensive literature search was conducted using Cochrane library, PubMed/MEDLINE, and Embase databases with keywords like “interdental papilla,” “hyaluronic acid,” and “human”. Change in BT mean height (mm) was considered as the primary outcome while percentage of change in BT area was considered as the secondary outcome.

**Results:**

177 articles were screened, and 15 eligible studies were included, focusing on the therapeutic effects of HA injections on interdental papilla dimensions in humans. Clinical trials have demonstrated varying degrees of success and patient satisfaction with HA injections for IDP reconstruction over a period of 4 weeks to 25 months. Several studies showed significant improvements related to BT height and width, although complete papilla fill remains unpredictable. Higher success rates were observed in the maxilla compared to the mandible, and patients with thicker gingival phenotype showed better outcomes. The initial size of the defect, the number of HA applications and the analysis method significantly influenced the results.

**Conclusion:**

HA injections look promising for IDP reconstruction. However, the need for multiple injections and long-term efficacy remains to be fully understood. Further research is necessary to standardise treatment protocols and evaluate long-term outcomes and patient satisfaction comprehensively.

Loss of interdental papilla (IDP) is a significant concern for both dentists and patients, driving an increased demand for treatment, as the presence of black triangles (BT) is considered the third most disliked esthetic problem below caries and apparent crown margins.^10^ The origin of BTs is multifactorial and can result from periodontitis, orthodontic treatment, crown and root shape and angulation, or interproximal contact position.^4^ Indeed, it was demonstrated that the distance from the contact point to the bone crest is key in IDP maintenance over time, and if this distance exceeds 5 mm, the percentage of proximal papilla presence decreases significantly.^38^ To address this esthetic issue, various multidisciplinary and invasive treatments have been proposed, including surgical, prosthetic, or restorative procedures.^31^ Non-surgical treatments, such as orthodontic treatments, have also been explored.^4^ Additionally, methods like papilla-sparing techniques during surgical interventions and use of soft tissue grafts have shown effectiveness in preserving or reconstructing IDP.^8^ However, such procedures are technically demanding and require skills and experience of the periodontist.^9^ More recently, hyaluronic acid (HA) has been shown to improve periodontal treatment outcomes^12^ and could be used as adjunct to plastic surgery to treat gingival recessions.^34^ HA is a naturally occurring glycosaminoglycan found in connective, epithelial, and neural tissues. Its application in dermatology and cosmetic procedures, such as facial wrinkle reduction and lip augmentation, has been well-documented. Indeed, HA offers structural support to the extracellular matrix in the dermis, promoting fibroblast activation and facilitating the synthesis and deposition of collagen.^28^ These properties make HA an attractive option for dental applications aiming to restore the volume and contour of lost IDP. The injections of HA have emerged as a minimally invasive alternative to surgical procedures.^19^

Papilla reconstruction through HA injection typically begins with the administration of local anesthesia at the treatment site. Once anesthesia is achieved, the anesthesia cartridge is replaced with a cartridge containing HA. Various injection techniques for HA application are described in the literature.^6,20^ While some authors do not specify the exact method used,^23^ others provide detailed protocols.^20^

For example, some studies recommend injecting HA 3-4 mm apical to the tip of the papilla. After the injection, massaging the papilla to a coronal direction with a finger for one minute is suggested to facilitate HA penetration into the tissues beneath the papilla.^6^

Another commonly described technique is the “Three-Step Technique”.^14^ This approach involves three sequential injections:

The first injection is made above the mucogingival line to create an HA reservoir.
The second injection is administered just below the base of the papilla.
The final injection is delivered 2-3 mm apical to the tip of the papilla.

Many authors emphasise that the needle should be angled at 45 degrees toward the papilla during the injection. The volume of HA injected varies considerably across studies and is not standardised.^13^

One advantage of HA injections is their relative simplicity, as they can be performed using a standard syringe typically used for local anesthesia. This makes the procedure widely accessible and does not require specific instruments.

The aim of this systematic review was therefore to assess the scientific relevance of using HA to repair IDP loss in humans based on BT reduction and patient related outcomes. Additionally, it aimed to provide general guidance to help clinicians select the best option for IDP treatment and enhance clinical outcomes, with a particular focus on offering practitioners insight into the prognosis of papilla reconstruction through HA injections.

## Materials and Methods

### Eligibility Criteria

Case-control, cross-sectional, cohort or randomised controlled clinical trials, conducted on human subjects older than 18 years, were included in this review to evaluate the impact of HA injection for IDP reconstruction. A study was considered eligible for inclusion if it met the following criteria: 1) article written in English; 2) studies evaluating the therapeutic effect of HA injection on IDP dimensions in humans. Systematic reviews, meta-analysis, opinions, editorial letters, and conferences were excluded.

### Information Sources

Studies were accessible via several electronic databases, including the Cochrane Library, PubMed/MEDLINE, and Embase. All studies included in this review were published up until January 17, 2025.

### Search Strategy

A literature search was conducted according to PRISMA guidelines.^26^ The database screening was performed using the following search equation: ((interdental papilla) OR (papilla) OR (papilla defects)) AND ((hyaluronic acid) OR (crosslinked hyaluronic acid)) AND (human).

### Selection Process

After removing non-relevant articles by screening titles and abstracts, the full-text articles were obtained and evaluated separately by independent reviewers (AL, P-YG). In case of disagreement between reviewers, inclusion or exclusion of the articles was determined by a third reviewer (OH).

### Data Selection Process

The investigators (AL and P-YG) extracted data from the included articles and organised them according to the following criteria: author’s name, number of patients included, number of sites treated, inclusion criteria, exclusion criteria, type of papilla recession, site treated, injection protocol, composition of HA gel used (or brand), evaluated parameters, evaluation method, and follow-up.

### Effect Measure

Change in BT mean height (mm) was considered as the primary outcome while percentage of change in BT area was considered as the secondary outcome. The effect measure in terms of IDP height augmentation, or IDP reconstruction rate (IPRR) was also considered. Patient satisfaction was also assessed.

### Synthesis Methods

This review assessed the estimated percentage change in BT area and the reduction in mean BT height, expressed as the mean difference between baseline measurements and those taken at least three months after the HA injection protocol.

### Reporting Bias Assessment

The researchers (AL and P-YG) performed the quality evaluation of all included studies, and any divergences were resolved after discussion. Risk of bias was evaluated independently by each reviewer through a process of quality analysis according to the Cochrane Reviewers’ Handbook, and RoB2 tool initially proposed by Higgins et al.^15^ Risk of bias for non-randomised clinical trials was assessed with ROBINS-I tool.^37^ Plots representing the different risk of bias assessment were generated by Robvis visualisation tool.^22^ Any disagreements were resolved after discussion.

## Results

### Study Selection

**Fig 1:**
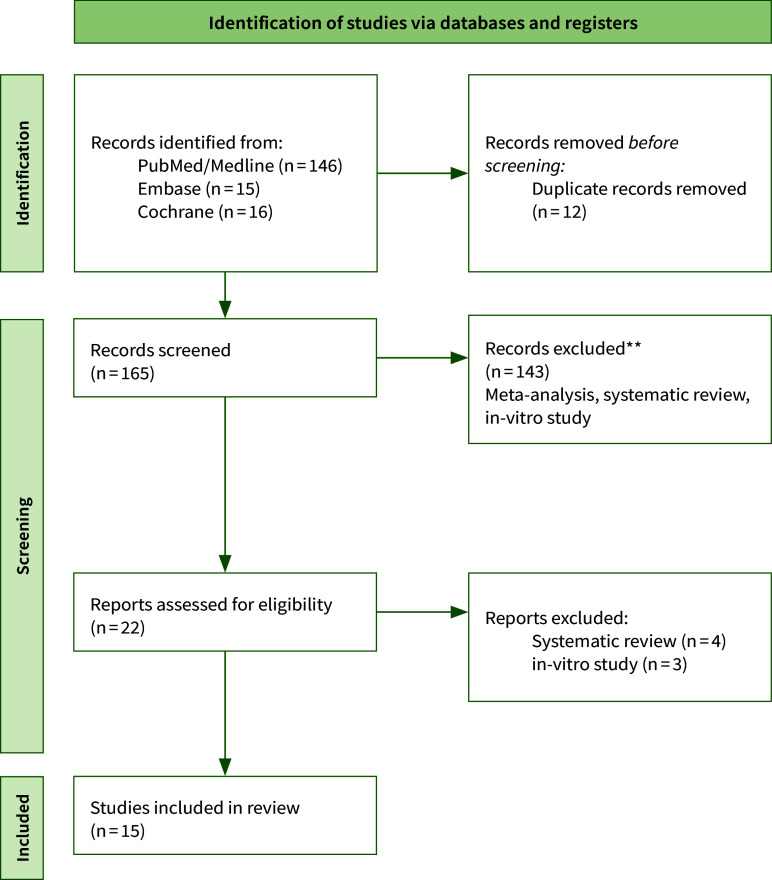
Search strategy flowchart.

The search strategy identified 177 potentially relevant publications. After screening of titles and abstracts, inappropriate papers were excluded resulting in 22 publications. As depicted in the PRISMA flow diagram (Fig 1), seven articles were excluded after full reading yielding fifteen articles included in this review according to the inclusion/exclusion criteria comprising four randomised clinical trials^1,7,20,24^ and eleven clinical trials.^2,6,8,17,18,21,23,32,35,36,39^

### Risk of Bias

**Fig 2:**
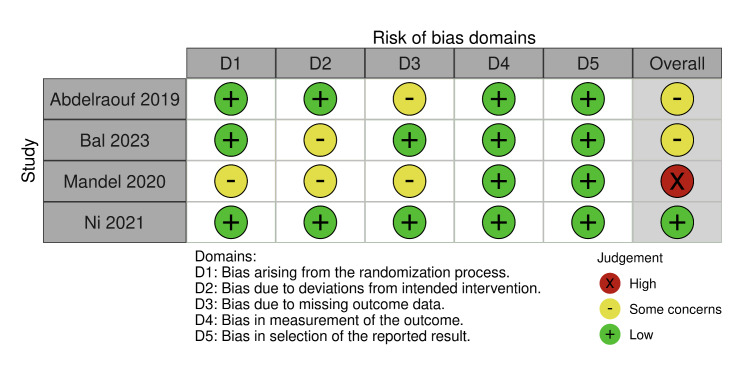
Risk of bias assessment for randomised clinical trials.

**Fig 3:**
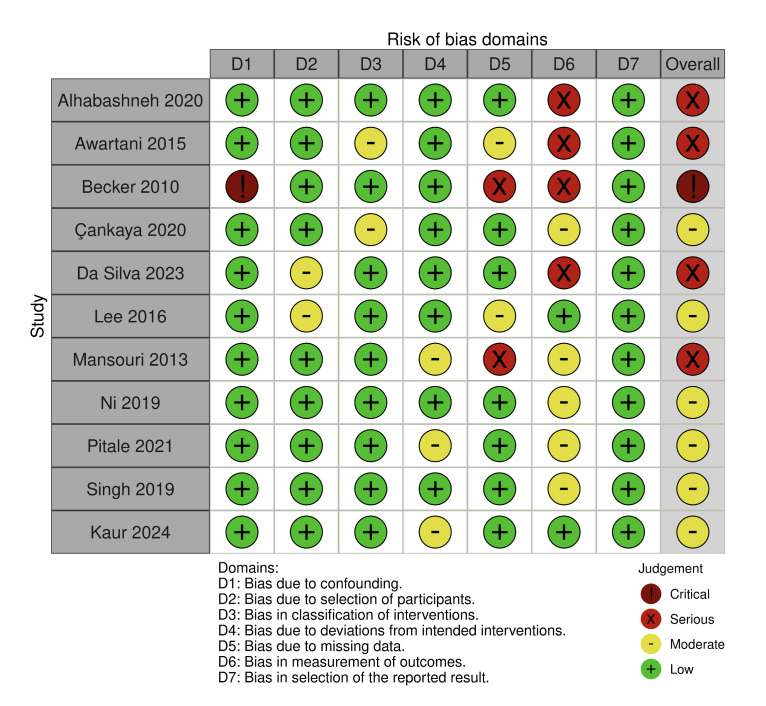
Risk of bias for non-randomised clinical trials.

The risk of bias assessment was conducted for the four identified randomised clinical trials,^1,7,20,24^ evaluating bias due to randomisation (D1), bias due to deviations from intended intervention (D2), bias due to missing data (D3), bias due to outcome measurement (D4) and bias due to selection of reported result (D5) (Fig 2). A risk of bias assessment was also performed for the eleven non-randomised clinical trials.^6,8,18,21,23,32,35,36,39^ evaluating bias due to confounding (D1), bias due to selection of participants (D2), bias in classification of intervention (D3), bias due to deviation from intended interventions (D4), bias due to missing data (D5), bias in measurement of outcomes (D6) and bias in selection of the reported result (D7) (Fig 3).

### Characteristics of Studies

The characteristics of the included studies are described in Table 1.

**Table 1:** Studies characteristics

Study	Number of patients	Number of sites	Inclusion criteria	Exclusion criteria		Papilla treated	Site treated	Injection protocol	HA gel	Parameters evaluated	Evaluation method	Follow-up
Abdelraouf (2019)	10 (3 males, 7 females)	30	Highly motivated patients Distance interproximal bone crest/contact point ≤ 7mm PPD ≤ 4mm at the deficient site PI/GI 0 to 1 No open contacts, caries, proximal restauration, fixed prosthesis or OT at affected teeth	Medical conditions that can affect periodontal healing or regeneration History of allergic reaction Pregnant or breastfeeding females Smokers Alcoholics Current or previous drugs intake that may predispose to gingival enlargement Under OT or OT in the past 6 months History of traumatical oral hygiene measures Periodontal surgeries over the last 6 months at the area of interest		Class I/II papilla recession (Nordland and Tarnow classification)	Interbicuspid region	Local anesthesia + 1 inj/IDP 0.1ml HA at J0/baseline, 3 weeks, 6 weeks) 2–3mm apical to the tip of the IDP	Restylane Lidocaine	BT height and area	Graduated periodontal probe + customised stent + standardised digital clinical photographs / image analysis program at 3 and 6 months	6 months
Alhabashneh (2020)	21 (17 females, 10 males)	86 (57 class I, 29 class II)	Caucasian Non smoking > 18 years old Distance alveolar bone crest/contact point ≥ 5mm No active periodontal disease Good OH	Spacing/crowding between teeth treated Abnormal tooth shape Systemic disease Pregnant and lactating women Tobacco users		Class I/II papillary recession (Nordland and Tarnow classification)	Central incisor, lateral incisor or canines Maxillar: 58 Mandibular: 28	Local anesthesia + 1 inj/IDP 0.2ml HA ; repeated at 21 days TST method	Hyadent BG	BT height	UNC-15 probe + digital photographs / analysis software at 3 and 6 months	6 months
Awartani (2015)	9 females	17	≥ 18 years old Systemically healthy	History of allergic reaction to injectable filler Smoking Pregnancy and lactation Medications affecting the gingiva or wound healing Periodontal surgery in the last 12 months Carious lesions or fixed restorations on study teeth Periodontitis Poor plaque control (visible plaque present, FMPS >20%)		Class I/II papilla recession (Nordland and Tarnow classification)	Anterior site (13 maxillary, 4 mandibular)	Local anesthesia + 1 inj/IDP 0.2ml HA at 2–3mm apical to the tip of the papilla; repeated at 21 and 42 days	–	BT area	Digital clinical photographs at 4 and 6 months	6 months
Bal (2023)	21 (10 males, 11 females)	34 sites	Patients with esthetic concerns; complaining of food lodgment in the anterior embrasure; with Class I and Class II papillary loss (Nordland and Tarnow); having adequate width of attached gingiva; within age group of 18–45 years; with plaque index <1 (Turesky, Gilmore and Glickman Modification of Quigley Hein 1970); and gingival index <1 at the involved sites (Loe and Silness 1967). The sites with a distance ≤7 mm from the interdental contact point to the interproximal bone crest and a probing depth of ≤4 mm at the defective papillary sites	Patients who received radiotherapy, chemotherapy, immunosuppressive treatments, systemic corticosteroids and/or anticoagulants the 30 days prior to intervention; history of allergy, systemic or blood borne diseases; prolonged treatment with non-steroidal anti-inflammatory drugs (NSAIDs) or similar medications; smokers; lactating or pregnant females; presence of composite and prosthetic restoration in maxillary anterior region; undergoing orthodontic treatment; having high frenum attachment; having midline diastema; and having any inability to take part in the investigation and comply with the required follow-up procedures. Sites with Nordland and Tarnow Class III papillary loss, sites with underlining intraosseous defects and implant sites		Class I/II papilla recession (Nordland and Tarnow classification)	Anterior sites (maxillary)	Local anesthesia + 1 inj/IDP with 0.2 ml of 0.8% hyaluronic acid gel or with 0.2 ml of 0.8% HA gel followed by 0.2 ml PRGF. 2–3 mm apical to the tip of the interdental papilla Repeated at 3 and 6 weeks	Gengigel (Ricerfarma) (0.8% gel)	BT height and area	Vernier caliper (bucco-palatal volume) + digital clinical photographs	12 weeks
Becker (2010)	11 (7 males, 4 males)	14 (10 implants, 4 teeth) *	Deficient papilla adjacent to teeth or implants	-		-	Esthetic area	Local anesthesia + < 0.2ml HA at 2–3 mm to the coronal tip of the IDP ; repeated up to 3 times (every 3 weeks)	Restylane	BT area	Photographs (no standardisation); computer program measured changes in pixels	From 6 to 25 months
Çankaya (2020)	20	200	Interdental embrace between natural teeth No systemic disease Non smoker Not pregnant or lactating No diagnosis of periodontitis No use of antibiotics or drugs that may affect the periodontal tissue in the last 3 months No prosthetic restoration in the maxillary or mandibular anterior region 5 interdental papillary spaces adjacent to each other in the inter canine-canine region Distance ≤ 5mm between alveolar crest and interproximal contact point	No contact point Insufficient plaque control Pocket depth of > 3mm in the treated areas < 2mm keratinised gingiva		MPIS = 2 (modified papilla index score)	Inter canine-canine spaces (5 adjacent interdental papillary spaces) on maxillary + mandibula	Local anesthesia + 3 injections/IDP (around 0.1ml for each IDP) Injections repeated at 3 weeks +/– third session if needed	Hyadent BG	Area of interdental space at 3, 12 and 24 months	Digital impression before and at 3, 12 and 24 months after treatment + image analysis program	2 years
Da Silva (2023)	6 (4 males, 2 females)	19	At least 1 defective papilla between 2 teeth, 1 teeth and 1 implants or 2 implants ** No significant systemic disease Non smokers	Smokers Significant systemic diseases		–	Maxillary anterior central and lateral incisors	Local anesthesia + <0.02ml injection 2–3mm above the papilla tip at 45° (until overflow into the sulcus) + application of solid petroleum jelly 3 injections with 4-week intervals	Rennova Fill HA gel (Innovapharma)	Papilla height + BT area	Standardised photographs (fork with acrylic resin) + ImageJ software linear analysis + Intraoral scan (Cerec Omnicam, Dentsply Sirona)	4 months
Kaur (2024)	15	15	Subjects aged 19-50 years old with at least one class I (Nordland and Tarnow) deficient papillary site and plaque index (Silness and Loe) and gingival index (Loe and Silness) scores between 0 and 1 were finalised for the study	Subjects with smoking and tobacco chewing habits were excluded from the study. Those having periodontitis or periodontal pocket >4 mm, gingival enlargement, autoimmune diseases, systemic diseases, and conditions (e.g., chronic inflammation, hepatitis, rheumatoid arthritis, atherosclerosis, and diabetes mellitus), allergy to any of the medications or history of food allergy were excluded from the study. Pregnant and lactating mothers were also excluded. Subjects with <2 mm of keratinised gingiva and fixed restorations on study teeth were excluded		Class I (Nordland and Tarnow)	Anterior aesthetic zone	Local anesthesia was administered 2–3 mm apical to the tip of the papilla (0.2ml)	Aqua Plus Cross Linked Hyaluronic Acid Dermal Filler	BT area + BT height	Digital clinical photographs	4 weeks
Lee (2016)	10	43	Adults patients At least 1 IDP deficiency with the presence of a contact point between adjacent teeth in the maxillary anterior region Plaque and gingival index between 0 and 1	Pregnant Medication known to increase the risk of gingival hyperplasia OT in the maxillary anterior region		–	Maxillary anterior region	1 point injection 2–3mm to the tip of the IDP (<0.02ml) Repeated up to 5 times during 3 weeks intervals	Teosyal Purescence Global Action	BT area	Standardised photographs + software analysis	6 months
Mandel (2020)	40 (30 females, 10 males)	160	At least 2 upper and 2 lower IDP	Active periodontitis (CPI 3 or 4) Acute oral and/or upper respiratory tract infection Previous surgical treatment of the papillae to be investigated Pregnancy or lactation Smoking Bleeding disorders or any medication that would affect blood coagulation Systemic diseases that may affect periodontal health Known or suspected allergy to local anesthetics and/or HA		Class I/II papilla recession (Nordland and Tarnow classification)	Front region between canine	TST method (0.3ml)	Flex Barrier or Regident	BT area	Photographs + imaging software for analysis (immediately after injection, at one week and one month)	1 month
Mansouri (2013)	11	21 (16 females, 5 males)	20–75 years old Possession of maxillary anterior teeth Plaque index < 20% Teeth free from caries with no fixed prosthesis or orthodontic appliance Non-smoker No history of systemic disease affecting the periodontal status No consumption of drugs causing gingival hyperplasia	-		Type I (86%) or III (Nordland and Tarnow classification)	Esthetic area	Local anesthesia + <0.2ml HA 2-3mm to the coronal tip of the papilla Repeated at 3 weeks, 3 months and 6 months	-	BT area	Clinical photographs + image J software analysis	6 months
Ni (2019)	8 females	22 (17 maxillary, 5 mandibular)	20–60 years old No systemic disease Good oral hygiene (FMPS <20% At least one IDP loss on the anterior maxilla or mandible Healthy periodontal tissue or inflammation controlled	Fixed prosthesis or carries on the studied teeth No contact point on the studied teeth History of allergic reaction to injectable filler Periodontal surgery in the last 6 months Regular medication intake affecting the gingiva metabolism		Class I/II papilla recession (Nordland and Tarnow classification)	Anterior maxilla or mandible	Local anesthesia + injection at the base of the papilla (0.05 to 0.1ml at each site), repeated at 3 and 6 weeks	Qi sheng biological agent company Limited	Height of gingival papilla and BT area	Clinical photographs before treatment and at 3, 6 and 12 months + software analysis	12 months
Ni (2021)	24	68	20–70 years old No systemic disease that would affect periodontal treatment No fixed prostheses or caries on the studied teeth Good oral hygiene (FMPS <20%) 2 or 4 symmetrical gingival papillary defects class I or II* in the anterior maxilla Healthy periodontal tissue or well-controlled inflammation (<10% bleeding sites with probing depth ≤3mm) No history of periodontal surgery in the last 6 months No smoking No history of regular medication intake that could affect gingiva metabolism			Class I/II papilla recession (Nordland and Tarnow classification)	Anterior maxilla	Local anesthesia + injection at the base of the papilla (0.05 to 0.1ml at each site), repeated at 3 and 6 weeks	Qi sheng biological agent company Limited	Height of gingival papilla and BT area + proliferation and migration of gingival fibroblasts in vitro	Clinical photographs before and at 6 and 12 months	12 months
Pitale (2021)	7 (2 males, 5 females)	25	Good health No radiographic evidence of interdental bone loss Pocket depth ≤ 4mm Good plaque control Tooth mobility score 0	Systemic disease Blood disorders Pregnant or lactating women Tobacco users		Class I/II papilla recession (Nordland and Tarnow classification)	Esthetic areas	0.2ml of HA injected 2–3mm apical to the coronal tip of papillae	2% injectable HA filler (Genoss)	BT height and width	Clinical assessment (probe + prefabricated standardised acrylic stent) + photographs and image analysis software At 3 and 6 months	6 months
Singh (2019)	10	42 (1% HA 16 sites, 2% HA 14 sites, 5% HA 7 sites)	25–40 years old Clinically normal periodontium PPI score 2 and 3 FMPS <10%	Known allergy to hyaluronic acid Poor plaque control Medically compromised Teeth with hopeless prognosis Parafunctional habits Traumatic occlusion Periodontal plastic surgery during the last 1 year Adjacent teeth with caries, fixed prosthesis or OT Drug-induced gingival overgrowth Pregnant and lactating women Smokers		PPI score 2 or 3 (papilla presence index)	Anterior maxillary (17 sites) or mandibular (18 sites)	Local anesthesia + HA injected 2mm apical to papillary tip (<0.2ml) Repeated on 2nd and 3rd weeks	1%, 2% and 5% HA gels	Clinical measurement: linear measurement from contact point to tip of the papilla + BT area	Clinical analysis: UNC-15 probe and modified stent at 1, 3 and 6 months + photographic analysis with imaging software	6 months
IDP: interdental papilla; BT: black triangle; MPIS: modified papilla index score; PPI: papilla presence index; TST: three steps technique; inj: injection. OT: orthodontic treatment. * Only results at tooth sites were analysed. **Authors did not distinguish interdental papilla between teeth, interdental papilla between implant and tooth, and interdental papilla between implants.

#### Observation period

Over the thirteen studies retrieved,^1,2,6,8,18,20,21,23,24,32,35,36^ the authors evaluated the effect of HA injection over periods ranging from four weeks^17^ to 25 months.^8^

#### Techniques

In most studies, the protocol for HA injection into the IDP was only briefly described.^1,6,8,17,18,21,36,39^ While many studies did not specify any particular technique, they consistently reported that HA was injected at a distance of 2–3 mm from the tip of the papilla.^1,6,8,17,18,21,32,35,36^ However, Ni et al^23^ did not provide details on the injection protocol, mentioning only that HA was injected at the base of the papilla, a detail also reported by Ni et al^24^ and Turgut Çankaya et al.^39^

Furthermore, Bal et al,^7^ Da Silva et al^35^ and Pitale et al^32^ detailed that the injection was made 2-3 mm from the papilla tip with a 45-degree needle angle.

Some studies employed a more specific technique, such as the “three-step technique,” which was previously described in the introduction.^2,20^

#### Volume injected, number of injections and intervals

The HA volume injected in the papilla varied greatly among the studies, from 0.002^18^ to 0.3 ml.^20^

The frequency and interval of HA injections varied significantly across the studies. In most cases, HA was injected between two and three times. In one study, two injections were administered^2^ while in nine studies three injections were administered.^1,6–8,21,23,24,35,36^ However, in one study by Lee et al,^18^ the injection was repeated up to five times. On the other hand, Mandel et al,^20^ Pitale et al^32^ and Kaur et al^17^ administered only a single HA injection.

The intervals between injections also differed greatly. In the majority of studies, injections were repeated every three weeks.^1,2,6–8,18,23,24,39^ Da Silva et al^35^ opted for injections every four weeks, while Mansouri et al^21^ administered injections at baseline, three weeks, three months, and six months. Singh et al^36^ employed a more frequent schedule, repeating injections every week.

#### Type of HA used

Various types of HA and concentrations were used across studies. Turgut et al,^39^ Alhabashneh et al,^6^ and Awartani et al^2^ used the same product, a combination of non-cross-linked HA (2 mg/ml) and cross-linked HA (16 mg/ml) in their trials. Similarly, Ni et al^23,24^ used the same HA formulation in both their 2019 and 2021 studies.

In contrast, only one study did not specify the type or reference of the HA used.^21^ The composition of HA fillers differed considerably depending on the brand, with some studies using reticulated HA.^2,6,39^ others employing crosslinked HA,^17,20^ and some using HA fillers combined with lidocaine.^1^

The concentration of HA was not consistently reported across studies. When it was specified, the concentration ranged from 0.8% 7 to 5%.^36^ In most studies, commercially available, ready-to-use injectable HA fillers were used. However, in the study by Singh et al,^36^ the researchers prepared different HA concentrations themselves, ranging from 1% to 5%, using HA powder.

#### Type of defects

Among the selected studies, only two did not mention the type of defect.^8,35^ In most cases, the authors characterised papilla deficiency using the Nordland and Tarnow classification,^18^ including only class I and II papilla deficiencies.^1,2,6,7,20,23,24,32^ Kaur et al^17^ included only class I Nordland and Tarnow papilla deficiencies, while Mansouri et al^21^ included class I and III. Meanwhile, Turgut Çankaya et al^39^ and Singh et al^36^ opted to include class 2 Modified Papilla Index Score (MPIS) papilla deficiency^39^ and class 2 or 3 Cardaropoli Papilla Presence Index (PPI),^36^ respectively.

#### BT reduction

Several studies have analysed the effect of HA injections on black triangle (BT) mean height.^2,17,18,23,32,35,36^ The results varied statistically significantly, with changes in BT height ranging from 0.21 ± 0.6 mm^36^ to 1.1 ± 0.35 mm^32^ after three months. At six months, the changes ranged from 0.16 mm^37^ to 1.06 ± 0.33 mm.^33^ Pitale et al^32^ demonstrated a significant improvement at six months following a single HA gel injection, showing a statistically significant increase in both BT height and width from baseline to three and six months (p = 0.01). However, no statistically significant difference in BT mean height was observed between three and six months (p ≥ 0.05), indicating that the results remained stable up to six months following the single injection.^32^ Singh et al^36^ compared the efficacy of three HA concentrations (1%, 2%, and 5%) and found that the 5% HA gel resulted in highly significant BT mean height improvement (p = 0.001). Both 1% and 2% HA gels significantly improved the BT mean height, although their effects were less significant compared to the 5% gel.^36^

Similarly, Alhabashneh et al^2^ found that HA injections produced promising results over the first six months, with the most significant BT reduction observed at three months. A slight reduction in improvement occurred between the three- and six-month marks. The study also showed a significant difference in overall BT reduction between maxillary and mandibular treated sites (p < 0.001), with greater improvements seen in the maxilla. However, there was no statistically significant difference between class I and class II papillary sites (p > 0.05) based on the Nordland and Tarnow classification.^2^

In 2023, Da Silva et al^35^ observed no statistically significant change in BT height following HA injection when analysed through photographic methods. However, the CAD/CAM analysis revealed improvements in BT height at three and four months compared to one month, with reductions of 0.41 ± 0.21 mm and 0.38 ± 0.21 mm, respectively (p < 0.0001). These findings highlight the importance of using precise analysis methods, such as CAD/CAM, to evaluate BT reduction accurately.^35^

Some studies also assessed changes in BT area. The BT area was reduced from 29.52 ± 18.72%^21^ to 58 ± 32.9%^35^ at three months and from 41 ± 16%^6^ to 92.55 ± 13.46%^18^ at six months. Awartani et al^6^ demonstrated statistically significant differences in BT area between baseline and four to six months (p < 0.0001), although no statistically significant differences were found between the four- and six-month periods (p > 0.12). A complete fill of the lost papilla remained uncommon, with less than 20% of treated sites achieving complete fill, even after three injections.^6^

Turgut Çankaya et al^39^ found similar trends, with better papilla filling percentages in the maxilla at three and twelve months compared to the mandible. The study suggested that improvements could be achieved more rapidly and effectively in the maxilla, a result that aligns with Alhabashneh’s findings. Additionally, Turgut Çankaya et al^39^ were the first to inject HA into five adjacent interdental papilla gaps and assess its efficacy using digital impressions over a two-year follow-up. The highest percentage of improvement was observed between the canine and lateral incisor sites, indicating that the effectiveness of HA injection may depend on the site being treated.^39^

Da Silva et al^35^ corroborated these findings, showing a mean papilla gain of 58% at three months. However, a relapse was observed at four months, with the papilla gain decreasing to 49.1% compared to baseline.

Finally, changes in BT mean area (in mm²) were also evaluated. Ni et al^23^ reported a significant reduction in BT area at three months, with a decrease of 0.31 ± 0.46 mm^2^.^23^ At six months, Lee et al^18^ and Ni et al^24^ found improvements in BT area of 0.21 ± 0.14 mm^2^ and 0.41 ± 0.56 mm^2^, respectively. In 2019, Ni et al^23^ further examined clinical outcomes based on gingival thickness and found a significant increase in BT height and decrease in BT area between baseline and three or six months, but only for patients with a thick gingival phenotype. Notably, this improvement was not statistically significant at twelve months, nor at any time point in patients with a thin gingival phenotype. These results suggest that HA injections may be more effective for patients with a thick gingival phenotype, and that repeated injections may be necessary after twelve months to maintain improvements, as relapse of the BT area can occur.^24^

Kaur et al^17^ also found a similar trend, but at a shorter timepoint comparing with the others studies. Indeed, they found an improvement of BT area changing from 0.54 mm^2^ ± 0.6 mm^2^ (p < 0.05) at baseline to 0.26 mm^2^ ± 0.30 mm^2^ (p < 0.03) at four weeks, occurring in a mean BT area change of 0.28 mm^2^ ± 0.3 mm^2^ (Table 2).

**Table 2:** BT reduction and change

Study	Number of applications	Interval between applications	% change in BT height	Change in BT mean height (mm)	% change in BT area	Change in BT mean area (mm2)	Follow-up
Alhabashneh (2020)	2	3 weeks	At 3 weeks: -8% At 3 months: -39% At 6 months: -29%	At 3 weeks: -0.17 At 3 months: -0.83 At 6 months: -0.62	-	-	6 months
Awartani (2015)	3	3 weeks	-	-	At 4 months: -62% At 6 months: -41%	-	6 months
Turgut Çankaya (2020)	1 to 3	3 weeks	-	-	At 3 months: -55.72% ± 6.61 At 1 year: -72.31% ± 4.64 At 2 years: -79.03% ± 4.98	-	24 months
Da Silva (2023)	3	4 weeks	-	Photographic analysis ; At 1 month: -0.08 ± 0.21 At 2 months: -0.04 ± 0.16 At 3 months: -0.08 ± 0.3 At 4 months: -0.22 ± 0.29 CAD/CAM analysis: At 1 month: -0.13 ± 0.08 At 2 months: -0.24 ± 0.14 At 3 months: -0.41 ± 0.21 At 4 months: -0.38 ± 0.21	CAD/CAM analysis: At 1 month: -30.41% ± 23.4 At 2 months: -39% ± 26.1 At 3 months: -58% ± 32.9 At 4 months: -49.1% ± 46.1	-	4 months
Kaur 2024	1	–	-	At 4 weeks: -0.63 ± 0.4	-	At 4 weeks: -0.28 ± 0.3	4 weeks
Lee (2016)	Up to 5 (mean: 3.42)	3 weeks	-	At 6 months: -0.71 ± 0.27	At 6 months: -92.55% ± 13.46	At 6 months: -0.21 ± 0.14	6 months
Mansouri (2013)	3	At 3 weeks + at 3 months	-	-	At 3 weeks: -3.38% ± 3.07 At 3 months: -29.52% ± 18.72 At 6 months: -47.33% ± 20.20	-	6 months
Ni (2019)	3	3 weeks	-	At 3 months: -0.311 ± 0.51 At 6 months: -0.45 ± 0.5 At 12 months: -0.4 ± 0.52	-	At 3 months: -0.31 ± 0.46 At 6 months: -0.41 ± 0.56 At 12 months: -0.36 ± 0.57	12 months
Singh (2019)	3	1 week	-	With 1% HA: At 3 months: -0.38 ± 0.14 At 6 months: -0.16 ± 0.08 With 2% HA: At 3 months: -0.21 ± 0.6 At 6 months: -0.21 ± 0.2 With 5% HA: At 3 months: -0.81 ± 0.21 At 6 months: -0.71 ± 0.21	-	-	6 months
Pitale (2021)	1	-	-	At 3 months: -1.1 ± 0.35 At 6 months: -1.06 ± 0.33	-	-	6 months
BT: black triangle; HA: hyaluronic acid.

#### HA Formulation and placebo comparison

Several studies compared different types of HA formulations,^20^ evaluated the efficiency of HA gel vs saline solution injections,^1,24^ or even compared the HA effect versus HA with an adjunct of Plasma Rich in Growth Factors (PRGF).^7^ Mandel et al^20^ compared the results at one month after injecting two different HA fillers and compared these with untreated sites. The untreated sites showed a slight increase in the black triangle (BT) area (+0.1% to +0.4%), whereas the HA fillers resulted in a reduction of the BT area by 4% to 13.9%, depending on the filler used.^20^

Bal et al^7^ compared the outcomes of HA and HA + PRGF at 12 weeks and demonstrated that both techniques were effective for papilla reconstruction, with the HA + PRGF group showing significantly better results.

Studies comparing HA fillers to saline solution injections also demonstrated that both treatments effectively reduced BT height. Abdelraouf et al^1^ reported that after three months, the BT height was reduced by 0.31 ± 0.25 mm in the HA group and by 0.07 ± 0.18 mm in the saline group. Similar findings were observed at six months, with reductions of 0.25 ± 0.26 mm to 0.198 ± 0.34 mm for the HA group and 0.03 ± 0.13 mm to 0.135 ± 0.39 mm for the saline group.

Furthermore, Ni et al^24^ demonstrated that both the HA and saline groups showed sustained improvements in BT height even after twelve months. The reduction in BT height was 0.280 ± 0.38 mm in the HA group and 0.278 ± 0.45 mm in the saline group^24^ (Table 3).

**Table 3:** Studies evaluating the effect of HA injection compared to a control group

Study	Group	Number of sites	Mean age	% change BT area	Change BT area (mm^2^)	Change in BT height (mm)	Follow-up
Abdelraouf (2019)	Test	16	32.55 ± 9.3	3 months: -36.5% ± 24.4 6 months: -45% ± 28.5	-	3 months: -0.31 ± 0.25 6 months: -0.25 ± 0.26	6 months
Control (saline)	14	3 months: -0.9% ± 10.6 6 months: -2.0% ± 11.4	-	3 months: -0.07 ± 0.18 6 months: -0.03 ± 0.13
Bal (2023)	HA	17	34.63 ± 5.22	12 weeks: -57.62 ± 21.78	-	-	12 weeks
HA + PRGF	17	38.71 ± 8.4	12 weeks: -77.42 ± 16.70	-	-
Mandel (2020)	Flex Barrier test	50	41.8 ± 13.8	1 month: -4% ± 8.8	-	-	1 month
Flex Barrier control (untreated)	30	1 month: +0.1% ± 0.9	-	-
Revident test	48	46.1 ± 12.3	1 month: -13.9% ± 22.6	-	-
Revident control (untreated)	32	1 month: +0.4% ± 4.6	-	-
Ni (2021)	Test	34	41.3 ± 7.73	-	6 months: -0.260 ± 0.42 12 months: -0.450 ± 0.54	6 months: -0.198 ± 0.34 12 months: -0.280 ± 0.38	12 months
Control (saline)	34	-	6 months: -0.150 ± 0.37 12 months: -0.320 ± 0.50	6 months: -0.135 ± 0.39 12 months: -0.278 ± 0.45
BT: black triangle.

Interdental papilla reconstruction rateSeveral authors expressed their results using metrics such as complete or partial interdental papilla reconstruction (CIPR/PIPR) and interdental papilla reconstruction rate (IPRR).^6,8,18,21,32^ Among the studies reviewed, the CIPR outcomes ranged from 18% 6 to 67%,^18^ while PIPR was achieved in 32% to 82% of cases.^6,18^

The percentage of sites with IPRR greater than 50% varied considerably, ranging from 43%^21^ to as high as 100%,^8^ illustrating the considerable heterogeneity in the results obtained.

Most of the studies focused on interdental sites, except for Becker et al,^8^ who compared the effects of HA injections at both tooth and implant sites. Their findings were promising, showing consistent improvements in both inter-dental and inter-implant papilla reconstruction. These improvements were sustained for up to 25 months, with an IPRR of over 50% at all sites.

Interestingly, Mansouri et al^21^ found a statistically significant relationship between age and papilla reconstruction success. Patients younger than 40 exhibited a mean papilla reconstruction improvement of 58.72% ± 20.84%, while older patients experienced a lower improvement rate of 34.80% ± 9.55% (p < 0.01) (Table 4).

**Table 4:** Studies evaluating the outcomes of HA injection in “interdental papilla reconstruction rate” (IPRR), in “complete interdental papilla reconstruction” (CIPR) or in “partial interdental papilla reconstruction” (PIPR)

Study	Number of sites	Number of applications	CIPR	PIPR	IPRR > 50%	IPRR < 50%	Follow-up
Awartani (2015)	17	3	3/17 sites (18%)	14/17 (82%)	8/17 sites (47%)	23%	6 months
Becker (2010)	4	2	0/4 sites (21%)	4/4 (100%)	4/4 sites (100%) IPRR from 76% to 96%	0%	6–25 months
Lee (2016)	43	Up to 5	29/43 (67%)	14/43 (32%) IPPR from 39% to 96%	-	-	6 months
Mansouri (2013)	21	3	-	-	43%	57%	6 months
Pitale (2021)	25	1	12/25 (48%)	13/25 (52%)	-	-	6 months


Patient satisfactionPatient discomfort following HA injections has been evaluated in several studies with varying results. In Alhabashneh’s study,^2^ 3 out of 21 patients reported mild pain during the first week after the injection, with significant relief from the third day. Notably, none of these patients required analgesics to manage the pain.^2^ In contrast, Awartani et al^6^ reported that 2 out of 9 patients expressed dissatisfaction with the procedure, specifically in terms of pain and discomfort, and only 66% of the patients indicated that they would undergo the procedure again.

Abdelraouf et al^1^ showed a statistically significant higher satisfaction score in the group receiving HA injections compared to the group treated with saline solution after six months.

In all three studies, local anesthesia was administered prior to the HA injection to minimise discomfort. However, differences in injection techniques and volumes used across the studies might explain the variations in patient satisfaction. For instance, while Awartani et al^6^ and Alhabashneh et al^2^ administered 0.2 ml of HA Abdrelraouf et al^1^ used a smaller volume of 0.1 ml. This difference in injection volume could potentially account for the lower satisfaction observed in the latter’s study compared to the others.

The data supporting the findings of this study are also available from the corresponding author upon reasonable request.

### Discussion

In recent decades, numerous clinical trials have evaluated the efficacy of hyaluronic acid (HA) injections in the correction of interdental papilla (IDP) defects. While promising results have been demonstrated for the reduction of black triangle (BT) height and area, the overall clinical relevance remains to be fully assessed.

The outcomes of HA injections for IDP defects have shown significant variability across studies. Complete interdental papilla reconstruction (CIPR) ranged from 18% 6 to 67% 19, while changes in BT mean height varied from 0.21 ± 0.6 mm^36^ to 1.1 ± 0.35 mm^32^ at three months, and from 0.16 mm^36^ to 1.06 ± 0.33 mm^32^ at six months. These findings indicate that while HA injections generally have a positive effect on BT reduction, the results are unpredictable and can vary widely.

One of the primary reasons for this variability is the heterogeneity in injection protocols used across different studies. The number of HA injections ranged from one to five,^17,20,32,18^ while the volume of HA injected varied from 0.002 ml to 0.3 ml.^18,20^ Furthermore, most studies did not adhere to a specific protocol, though some used a “three-step technique”.^2,20,23,24,39^ Injection intervals also varied widely, from once a week to once every three months.^21,36^ This inconsistency in protocols is likely to contribute to the variability of outcomes.

The rationale for repeated HA injections stems from HA’s structural properties and resorption rate. HA is commonly used in aesthetic medicine due to its ability to provide structural support by attracting water molecules, which increases tissue volume. However, HA’s effects are temporary, typically lasting four to six months.^40^ Given its well-documented use in aesthetic procedures, its application for treating papilla deficiencies was proposed. HA has been shown to enhance extracellular matrix remodeling and collagen maturation in gingival tissue^30^ by promoting fibroblast migration, proliferation, and viability.^5^

HA is also widely used in periodontology to improve healing following surgical treatments of infrabony defects. Onisor et al^25^ demonstrated that HA improves periodontal healing when used as an adjunct to open flap debridement (OFD), though their systematic review revealed high variability across studies. HA has similarly been used to enhance healing and attachment gain in mucogingival surgery, albeit with limited clinical improvement and significant heterogeneity in the studies reviewed.^33^ Recent research showed that cross-linked HA can positively impact dental biofilm by reducing the colony-forming unit count while simultaneously promoting periodontal healing through the stimulation of fibroblasts.^41^

HA injections for papilla reconstruction were also tested on rats. Pi et al^29^ evaluated the effect of HA injections for treating papilla deficiency. They found that the volume of the IDP was greater at sites treated with HA injections compared to those treated with phosphate-buffered saline (PBS) injections. Additionally, neo-microvascularisation was observed around the HA filler, without any signs of inflammation, demonstrating that HA injections is a reliable solution on IDP reconstruction.^29^

The variability in HA efficacy may be linked to the type of papilla defect, the site being treated, and the patient’s gingival phenotype. For example, Alhabashneh et al demonstrated that HA injections are effective for Nordland and Tarnow class I and II papilla defects,^2^ although no study has examined HA’s effects on class III defects. Additionally, the type of HA used in the studies varied, with some authors using cross-linked HA and others not specifying the HA’s composition or origin. This further complicates the ability to conduct a meta-analysis and draw definitive conclusions. To our knowledge, no study has been conducted on the impact of the composition of fillers used for the treatment of IDP deficiency. However, the composition and properties of HA should be taken into account, as factors such as HA molecular weight, crosslinking, concentration, and viscoelastic properties can have varying effects on the tissue.^16^

Several studies have suggested long-term improvements in papilla volume, even after a single injection,^32,40^ while others recommend multiple injections to stabilise results over time.^23^ Despite favourable outcomes of HA injection for IDP defects, the criteria for success still need to be established to predict clinical outcomes more effectively.

The lack of standardised evaluation methods, including the use of photographic analysis and CAD/CAM, further complicates direct comparisons between studies. Significant variations in results have been reported depending on the method of analysis, as demonstrated by Da Silva et al,^35^ suggesting that outcomes may be sensitive to the tools and measurements adopted.

The findings of this study align with previous systematic reviews that evaluated the effects of HA injections for papilla reconstruction. Our results indicate that HA injections significantly improved papillary reconstruction, with reductions in BTA ranging from 41%^6^ to 92.6%.^18^ This broad range is consistent with the findings of Makdisi et al^19^ and Ebrahimi et al,^11^ who reported improvements in BT area of 57.7% and 85.1%, respectively. These variations additionally underscore a degree of heterogeneity in the outcomes.

Our results align with those of Patel et al.^27^ However, the studies included in that review differ from ours, as some studies investigated papilla reconstruction using HA + PRF or employed the subperiosteal tunneling technique to enhance papilla laxity and achieve better BT reduction.^27^ Compared to the systematic review by Alsharif et al,^3^ we reached the same conclusion; however, we were unable to compare the degree of papilla reconstruction as their study did not provide any numerical data.

Despite the heterogeneity of the included studies, several general trends can be identified across studies:
Multiple injections are often required to achieve complete IDP recovery and to maintain stable results.
The most significant improvements occur within the first three months.
Higher and quicker improvements tend to be observed in the maxilla.
Smaller initial lesions show greater improvement.
A BT area up to 0.25 mm^2^, height up to 1 mm, and width up to 0.5 mm are largely associated with a 100% IPRR.
Younger patients (<40 years old) show better papilla reconstruction outcomes.

Patients with a thick gingival phenotype experience better results.

Patient satisfaction has generally been favourable, with minimal complications and moderate pain reported in the first week after injection. However, satisfaction requires more precise evaluation, as aesthetic expectations can vary greatly between patients and clinicians. Black triangles are often the primary reason patients seek treatment, and their expectations may not always align with clinical objectives.

This study shows certain limitations, as comparing results across studies is challenging due to variations in assessed parameters and differences in study designs. Another limitation of this review is that one of the included studies did not differentiate the results for IDP between teeth, between teeth and implants, or between implants.^35^ Long-term studies are needed to evaluate the durability of HA injections and to establish a standardised protocol for their use. Currently, there is no definitive evidence regarding the optimal HA formulation, concentration, or the best timing for repeated injections.

### Conclusion

Hyaluronic acid injections are emerging as a preferred treatment for reducing black triangles and enhancing IDP volume, with better results observed in the maxilla. Most improvements are achieved within three months, and repeated HA injections tend to yield better outcomes. Furthermore, smaller initial lesions and younger patients with a thick gingival phenotype show more favorable results. However, existing studies on HA injections for papilla reconstruction show statistically significant variations in methodology, underscoring the need for standardisation in both techniques and evaluation methods. Additionally, greater emphasis should be placed on assessing patient satisfaction.

### References
